# What Do We Know About the Preterm Behavioral Phenotype? A Narrative Review

**DOI:** 10.3389/fpsyt.2020.00154

**Published:** 2020-03-25

**Authors:** Grace C. Fitzallen, H. Gerry Taylor, Samudragupta Bora

**Affiliations:** ^1^School of Psychology, Faculty of Health and Behavioural Sciences, The University of Queensland, Brisbane, QLD, Australia; ^2^Mothers, Babies and Women’s Health Program, Mater Research Institute, Faculty of Medicine, The University of Queensland, Brisbane, QLD, Australia; ^3^Biobehavioral Health Centre, Abigail Wexner Research Institute at Nationwide Children’s Hospital, and Department of Pediatrics, The Ohio State University, Columbus, OH, United States

**Keywords:** ADHD, anxiety, autism, behavioral adjustment, high-risk children, neurodevelopment, phenotype

## Abstract

Preterm birth is associated with a significantly increased risk for childhood and adolescent psychopathology relative to full-term birth, with an inverse relationship between gestational age at birth and later risk for psychopathology. The manifestation of symptomatology and comorbidity profiles of emotional and behavioral adjustment problems in this high-risk group have been shown to be distinct from the broader pediatric population. Acknowledging these differences, a preterm behavioral phenotype has been proposed and increasingly recognized, highlighting the unique, frequent co-occurrence of symptomatology associated with attention-deficit/hyperactivity disorder, autism spectrum disorder, and anxiety disorders. The current state-of-the-art review provides a comprehensive characterization of this phenotype to date and further highlights key knowledge gaps primarily regarding the evolution of symptoms, co-occurrence of disorders and/or symptomatology within the phenotype, and associations of the phenotype with chronological age and degree of prematurity.

## Introduction

Preterm birth (<37 weeks gestation) affects approximately 15 million infants and their families around the world, annually, with vast improvements in survival reported in recent times (1). Two decades ago, the survival rate of infants born extremely preterm (EPT, <28 weeks gestation) was <75%, whereas in more recent years, this has increased substantially to >90% (2, 3). Preterm birth is now increasingly recognized as one of the most common risk factors for childhood psychopathology. Comprehensive research over the past three decades has profiled the neurodevelopmental consequences of preterm birth. Nonetheless, relative to our understanding of the motor and cognitive outcomes (4–6), robust conclusions regarding the true nature of childhood psychopathology remain a major challenge, spanning from subclinical to clinical presentations. To a reasonable degree, this ambiguity is due to substantial variability in outcome measures, an over-reliance on single informants, limited longitudinal investigations, and a primary focus on broad mental health classifications rather than clinical diagnostic criteria. It is also noteworthy that despite the disproportionate burden of preterm birth in Africa and other low-resource settings (7), existing studies reporting on psychopathology outcomes in this population are exclusively restricted to high-resource areas.

## Preterm Birth and Childhood Psychopathology

Over the past decade, there has been a general consensus that the landscape of childhood psychopathology in this high-risk population is changing. Investigations have intensified to support the existence of adverse behavioral and emotional consequences following preterm birth. Specifically, children and adolescents born preterm are at a two- to four-fold higher risk for a range of psychopathology relative to their full-term counterparts (8, 9). It is now also well recognized that the risk, severity, and extent of comorbid diagnosis of psychopathology increase with each decreasing gestational week at birth (8, 10, 11). Two recent meta-analyses synthesize findings on the prevalence of mental health diagnoses and symptoms in those born preterm compared to full-term (8, 12). The first meta-analysis investigated the prevalence of mental health disorders described in the *Diagnostic and Statistical Manual of Mental Disorders (DSM) Third Edition Revised (DSM-III-R)*, *Fourth Edition* (*DSM-IV*), or the International Classification of Diseases 10^th^ Revision (ICD-10) in children, adolescents, and young adults aged 10 to 25 years and born preterm or with low birth weight (LBW) compared to full-term peers (12). Studies were considered for inclusion if they were published between 1995 and 2010. Analyses of the five included studies confirmed that the rate of any psychiatric diagnosis in the preterm sample was significantly higher than that in the full-term sample. In addition, combined analyses of anxiety and depression found that the rate of these outcomes was also significantly higher in the preterm group (odds ratio 2.92, 95% confidence interval 1.82–4.67) (12). While the second meta-analysis investigated a similar age range and included studies published until 2016 and a broader range of mental health outcomes, it focused more exclusively on EPT or extremely low birth weight (ELBW) samples (8). From the 41 included studies, children (5–13 years) born preterm were at significantly higher risk for symptoms of the inattentive, hyperactive–impulsive, and combined subtypes of attention-deficit/hyperactivity disorder (ADHD), internalizing and externalizing behavior, conduct problems, social problems, and symptoms suggestive of autism spectrum disorder (ASD). Adolescents (14–18 years) were at significant risk for the three subtypes of ADHD and social problems, whereas adults (≥19 years) were only at risk for symptoms relating to anxiety, including internalizing behavior and shyness, indicating a relative reduction in risk across the developmental trajectory.

While both meta-analyses provide valuable insight into the mental health risks faced by individuals born preterm, they have similar limitations. First, the large heterogeneity of outcome measures included in the studies considered for these analyses make it difficult to compare scores across outcome domains. Most individual studies included in these meta-analyses had small samples sizes and focused on outcomes in younger age groups who are less likely to have received psychiatric diagnoses or experience clinically significant problems, with few investigating outcomes in late adolescence and adulthood. Finally, many of the studies included did not provide diagnostic details or conduct symptom-specific analyses. Nonetheless, these two meta-analyses confirm that preterm birth poses a major risk for the development of childhood psychopathology.

Despite these limitations, converging evidence suggests that the three most common psychiatric problems in children born preterm are symptoms and diagnoses of ADHD, ASD, and anxiety (13–16). A recent meta-analysis reported that those born very preterm (VPT)/LBW or EPT/ELBW were three times more likely to be diagnosed with ADHD compared to their full-term peers (17). This corresponds with the rates of ADHD diagnoses in the preterm population, ranging between 11% and 30% as reported across multiple cohort studies (10, 18, 19). Likewise, the rate of ASD in the preterm population is approximately 7% (4–9%), which is between two and three times higher than that in the general population (20–23). Finally, the rate of anxiety disorders is significantly higher in those born preterm compared to full-term peers, with a two- to three-fold increased risk (18, 24–26).

## The Preterm Behavioral Phenotype

In line with these findings, Johnson and Marlow reviewed commonalities in diagnostic studies of psychopathology outcomes in those born preterm (9). They identified a unique and consistent pattern of behavioral and emotional difficulties characteristic of ADHD, ASD, and anxiety disorders, conceptualized as the “preterm behavioral phenotype.” In addition to greater risk for these difficulties, their conceptualization of this phenotype also highlights differences in the etiology and presentation of symptoms. For example, in the those born preterm, ADHD is more likely to be associated with medical factors such as birth complications or brain injury/abnormalities compared to social factors and to manifest as the inattentive subtype of ADHD compared to the hyperactive–impulsive or combined subtype. Comorbidity with conduct and oppositional behavior is less common, and rates of the disorder are similar in males and females, as opposed to the greater prevalence of the disorder in males in the general population. In relation to ASD and related symptomatology, a recent systematic review (27) showed that children and adolescents born preterm are more likely to present with “autism-like symptoms” such as social withdrawal, social incompetence, and communication problems (28), or screen positive for ASD (23, 29), compared to rigorously defined diagnoses of ASD. Similarly, children born preterm are more likely to display internalizing behaviors that fall short of a clinical diagnosis of an anxiety disorder than to meet full diagnostic criteria (9).

Since the conceptualization of the preterm behavioral phenotype, there has been a growing body of research investigating the comorbidity of the three symptom clusters composing this phenotype. A concise summary of the preterm behavioral phenotype during the adolescent years was published in 2013 (28). The objective of this state-of-the-art review is to provide a comprehensive characterization of the preterm behavioral phenotype to date and highlight key knowledge gaps to inform future research, clinical practice, and policy.

## Search Method

In line with Preferred Reporting Items for Systematic Reviews and Meta-Analyses (PRISMA) guidelines (30), a systematic search was undertaken on PubMed/MEDLINE, PsycINFO, and the first 10 pages of Google Scholar electronic databases for peer-reviewed, original publications between January 1^st^, 2011, and January 31^st^, 2020, using the primary search terms “preterm behavioural phenotype” and “preterm behavioral phenotype.” To ensure comprehensiveness, a supplementary search was performed with the search terms “preterm” AND “phenotype” OR “preterm phenotype” AND “psychiatry” OR “psychiatric” OR “attention-deficit/hyperactivity disorder” OR “inattention” OR “autism spectrum disorder” OR “social” OR “social–emotional” OR “anxiety” OR “internalizing”/”internalising”, with one additional study identified meeting the inclusion criteria of this review. Studies were considered for inclusion in this review if they investigated the behavioral and/or emotional outcomes of children and adolescents born preterm, spanning across at least two of the three symptom clusters (e.g., ADHD and/or ASD and/or anxiety), and if they made explicit reference to the preterm behavioral phenotype in the background or discussion of the study when interpreting findings. Studies were excluded if they investigated only one of the three phenotype clusters or if they exclusively recruited a medically high-risk preterm sample (e.g., brain injury, small for gestational age, etc.), due to these groups being less representative of the larger preterm population. **Table 1** illustrates the sample characteristics of the seven studies summarized in this review, including country and year of birth, sample size, age at the time of assessment, proportion of males, and gestational age of the preterm and term-born comparison groups. Studies are reviewed in chronological order of the year of publication to reflect the evolving knowledge base of the phenotype.

**Table 1:** Sample characteristics of included studies investigating the preterm behavioral phenotype.

Reference	Country	Year of birth	Age at assessment, years	Preterm group			Full-term group		
				Sample size, n	Gestational age at birth, *M* (*SD*), weeks	Male, %	Sample size, n	Gestational age at birth *M* (*SD*), weeks	Male, %
Loe et al. (31)	USA	1992–1999	9–16	25	28·6 (N/R)	48	20	39·3 (N/R)	50
Taylor et al. (32)	USA	1992–1995	14	169	26·4 (2)	37	115	37 (N/R)	36
Korzeniewski et al. (33)	USA	2002–2004	10	776	<28 weeks*	N/R	–	–	–
Samuelsson et al. (34)	Sweden	1992–1998	10–15	130	24·4 (0·7)	46	103	39·4 (1·3)	51
Johnson et al. (35)	United Kingdom	2009–2010	2	638	34·9 (1·2)	54	765	39·3 (1·4)	50
Burnett et al. (36)	Australia	2005	8	181	26·8 (1·9)	57	185	39·8 (1·3)	56
Lean et al. (37)	USA	2007–2010	5	85	26·5 (1·8)	44	40	39·5 (0·8)	43

N/R, not reported. *No mean gestational age of sample reported.

## Narrative Review

Loe et al. (31) investigated the relationship between symptomatology across two of the three phenotype clusters, as well as externalizing behavior. They examined measures of white matter integrity to determine potential underlying neurobiology of the preterm behavioral phenotype. Parent reports were obtained on the Child Behavior Checklist (CBCL) for the three scales of internalizing (anxiety cluster), externalizing, and attention problems (ADHD cluster) in 25 preterm (mean gestational age 28.6 weeks) and 20 full-term children aged between 9 and 16 years from USA. Findings showed that mean inattention and internalizing scores were higher in those born preterm compared to full-term, with no significant group differences for externalizing behavior. They found that all three scales were highly correlated, especially the inattention and internalizing scales. Additional analyses showed that for the preterm group only, attention and internalizing problems were both significantly negatively associated with fractional anisotropy measures from diffusion tensor imaging, whereby more behavior problems were associated with lower fractional anisotropy in the forceps major and minor, inferior fronto-occipital fasciculus/inferior longitudinal fasciculus, and superior longitudinal fasciculus. These findings suggest associations of inattention and internalizing behavior problems with a widely distributed and overlapping network of white matter abnormalities. Social outcomes were not assessed in this study, precluding investigations of potential associations of social problems with inattention and internalizing behavior. A further limitation is that participants were recruited from a small convenience sample, limiting investigations of comorbidity in the broader population of preterm children.

Taylor et al. (32) is the first study to have explored emotional and behavioral outcomes across all three symptom clusters at both a dimensional and a categorical level in 169 ELBW adolescents and 115 normal birth weight peers at 14 years of age from a single center in Ohio, USA. Parents reported outcomes on the Adolescent Symptom Inventory-4, with self-reports also collected on the Youth’s Inventory-4. This is the only study summarized in this review that utilized self-reported behavioral and/or emotional outcomes of children born preterm. Based on parent reports, adolescents had significantly higher item ratings on measure domains (dimensional model) for inattentive ADHD, combined ADHD, generalized anxiety disorder, and social phobia compared to normal birth weight peers. The preterm group also had significantly higher rates of scores exceeding the clinical cutoff for inattentive ADHD and specific phobia, suggesting that the children met the diagnostic criteria for these disorders. Although the groups did not differ significantly in the presence/absence of at least one *DSM-IV* disorder, there were significantly more ELBW adolescents meeting criteria for three or more disorders (n=10 vs. 1). In contrast, ELBW adolescents self-reported lower scores on all measures compared to normal birth weight adolescents, with significant differences for item ratings of oppositional defiant disorder and rates of inattentive ADHD and conduct disorder. From ELBW self-reports, there were also no significant differences in the rates of one or more, or three or more, co-occurring disorders. While a strength of this study is the comparison between parent and adolescent reports, a limitation in the current context is the failure of the study to focus specifically on the co-occurrence of disorders within the phenotype. This information would have provided support for the proposed unique pattern of behavioral and emotional difficulties. Methodologically, findings may be further limited by reliance on clinical cutoff thresholds as applied to ratings rather than on clinical psychiatric diagnoses.

Korzeniewski et al. (33) described the prevalence of social impairment, as defined by elevated ratings on the Social Responsiveness Scale (SRS), in 776 children born EPT who did not meet criteria for an ASD diagnosis at 10 years of age, recruited through multi-center sites in USA. They further investigated the relationship between this social impairment and the remaining two symptom clusters of ADHD and anxiety. Approximately 80% of children had an intelligence quotient score ≥85; hence, the majority of the sample was of normal intelligence, with 16% identified as having SRS-defined impairment. When comparing scores on the SRS to other communication domains, those with SRS-defined impairment were more likely to demonstrate deficits in verbal communication, reciprocal social interactions, and repetitive behaviors on the Social Communication Questionnaire, as well as impairments in structural and pragmatic language. Children with SRS-defined impairment were more likely to screen positive for ADHD and display symptoms of generalized anxiety disorder, separation anxiety disorder, social phobia, major depression, dysthymic disorder, and/or oppositional defiant disorder/conduct disorder on the Child Symptom Inventory-4. Teacher reports on the Child Symptom Inventory-4 Teacher Checklist also showed associations between SRS-defined impairment and positive screens of inattentive ADHD and generalized anxiety disorder. Findings demonstrate that children born preterm are at risk for a range of social and communicative problems characteristic of ASD, and that those with social impairment are also more likely to have other symptoms characteristic of the preterm behavioral phenotype, with support shown for associations of ASD with ADHD and of ASD with anxiety-related symptomatology. However, the association between ADHD and anxiety remains unclear, as this relationship was not investigated in this study.

Following on from this, Samuelsson et al. (34) investigated the frequency and severity of behavioral and emotional problems in children aged 10–15 years born EPT using parent and teacher reports in Sweden compared to full-term peers. The preterm group consisted of 130 parents and 128 teachers and the full-term group of 103 parents and 103 teachers. Across both parent and teacher ratings, children born EPT had significantly higher ratings of internalizing, attention, and thought problems on the CBCL and Teacher Report Form compared to full-term peers. In addition, parents reported significantly higher social problems in the EPT group compared to full-term peers. The preterm group had higher ratings of ADHD symptoms on both the Conners’ Parent Rating Scale and Teacher Rating Scale, with scores more likely to be within abnormal ranges for restlessness, impulsive behavior, and emotional lability. Findings were similar when excluding children with neurodevelopmental disabilities. These findings support previous research, which has shown that those born preterm are at significant risk for the presence of ADHD-related symptoms and provide evidence for the comorbidity of parent-reported internalizing and social problems. A methodological strength of this study is that multiple informants were utilized to provide comparisons between parents and teachers; however, the study did not investigate comorbidity patterns between phenotype symptoms, only independent presence.

Unlike the previous studies reviewed above, Johnson et al. investigated the comorbidity of cognitive and behavioral outcomes in late to moderate preterm (LMPT) born toddlers in the United Kingdom (35). This is the only study included in this review that investigated an LMPT sample. In this study, 638 2-year-old LMPT children were compared to 765 term-born children on assessments of behavioral, social–emotional, and ASD-related problems, cognition, and language. Measures included the Brief Infant-Toddler Social and Emotional Assessment, the Modified Checklist for Autism in Toddlers, and the Parent Report of Children’s Abilities—Revised, respectively. Using latent class analysis, three distinct comorbidity profiles emerged. Class 1 was described as “optimal outcomes,” with an absence of cognitive and behavioral outcomes in both LMPT and term-born children. Class 2 was described as “non-optimal outcomes,” characterized by behavioral problems, delayed social–emotional competency, and a moderate risk for ASD symptoms and eating difficulties in both LMPT and term-born children in the context of normal-range cognition and language. As Class 3 consisted of only LMPT children, it was characterized as the “preterm phenotype.” Class 3 children displayed symptoms of ADHD along with substantial problems in cognition, language, and social–emotional competency. This study is the first systematic investigation to disentangle the proposed comorbidity of the preterm behavioral phenotype, suggesting that there may be more than one behavioral and/or cognitive profile seen in those born preterm, some of which may be shared with those born at term. While this study contributes valuable knowledge to the ongoing investigations of the phenotype and the risk that it poses to children born preterm, findings should be interpreted with caution related to the lack of clear criteria for selection of the three-profile model. Analyses of dichotomous variables (impaired vs. unimpaired) may have also limited the power of the study to detect specific cognitive and behavioral profiles.

Another investigation of the comorbidity of symptoms comprising a potential preterm behavioral phenotype was conducted by Burnett et al. (36). This study included Australian children born EPT/ELBW at 8 years of age (n=181) compared to term-born children (n=185). Behavioral and emotional outcomes were assessed using the Strengths and Difficulties Questionnaire (SDQ), with analyses revealing four distinct profiles. Profile 1 consisted of children with minor symptom problems across all domains from families of higher socio-economic status and included more girls than boys. The majority of the sample fell into this class and included 55% of the EPT/ELBW group and 74% of the term-born group. Profile 2 comprised children with subclinical elevations in emotional, hyperactive/inattentive, and peer-related symptoms and had the highest rate of major neurosensory disability. This profile most closely resembles the proposed preterm behavioral phenotype as described by Johnson and Marlow (9), though it included only 20% of the EPT/ELBW group. Profile 3 was characterized by higher emotional symptoms, conduct problems, hyperactivity/inattentiveness, and peer-related problems and included 16% of the EPT/ELBW group. Profile 4 included fewer cases than the other profiles, had more boys than girls and only 8% of the EPT/ELBW group, and was characterized by clinically significant (above threshold) median scores in all SDQ domains. Profiles 2, 3, and 4 consisted of higher “definite” behavioral problems on the SDQ and poorer numeracy and literary skills compared to Profile 1. The children classified into Profiles 3 and 4 also had lower than average intellectual functioning as measured on the General Conceptual Ability Index of the Differential Ability Scales-II. This study builds on that by Johnson et al. (35) by providing a symptom-based approach to profile analysis. Similar to other reports, this study was also limited by a lack of evaluation of the clinical severity of symptoms due to the screening nature of the primary outcome measure.

Maintaining the theme of cluster-based approaches, Lean et al. (37) identified four behavioral profiles in 5-year-old children born VPT (n=85) and full-term (n=40) in USA. Behavioral and emotional difficulties were measured using both parent and teacher reports on the CBCL (internalizing and externalizing), Conners’ Rating Scale—Revised (ADHD), and SRS (ASD). Clinical diagnoses were made using the Preschool Age Psychiatric Assessment and Autism Diagnostic Observation Schedule. Profile 1 grouped typically developing children comprising 39% of the total sample (VPT: 27%, full-term: 65%). These children exhibited neurodevelopmental and psychiatric levels similar to that in the general pediatric population. Profile 2, an at-risk group (38%; VPT: 44%, full-term: 23%), described children with lower neurodevelopmental scores and slightly elevated psychiatric profiles but remaining within the typical range. Profile 3 represented the psychiatric group (12%; VPT: 13%, full-term: 10%), including children with moderate–severe difficulties with executive function and ADHD and ASD symptoms seen through both parent and teacher report. Finally, Profile 4 characterized children into an inattentive/hyperactive group (VPT: 15%, full-term: 3%), classified by low cognitive and language scores and high levels of parent- and teacher-reported ADHD. In addition, teachers rated children in this group as notably higher in internalizing, externalizing, and ASD-related behaviors. The highest number of children born VPT were classified into the at-risk group showing slightly elevated yet typical psychiatric difficulties. In line with the preterm behavioral phenotype, significantly more children born preterm were categorized into the “inattentive/hyperactive group” compared to children born full-term (15.3% vs. 2.5%). While these findings indicate that preterm birth poses a greater risk for experiencing moderate–severe psychiatric symptoms characteristic of the phenotype at preschool age, it is recommended that these findings be interpreted with caution with consideration to the small sample size and discrepancy between parent and teacher reports.

## Gaps in the Preterm Behavioral Phenotype

Since the conceptualization of the preterm behavioral phenotype, seven studies have investigated two or three symptom clusters characteristic of ADHD, ASD, or anxiety, with explicit reference to the phenotype in the study rationale or interpretation of findings. Research has increased knowledge of the variable pattern of behavioral and emotional difficulties in the preterm population and of the co-occurrence of symptom clusters within the phenotype. Despite these advances, there are several methodological challenges that continue to hamper our knowledge in this area. The first challenge is the substantial heterogeneity of outcome measures administered in follow-up assessments of preterm samples. As shown in **Table 2**, different measures were utilized in each of the studies reviewed above, with the exception of some study overlap in the use of the CBCL, Conners’ Parent Rating Scale, and SRS. As a result, there is inconsistency and limited comparability of behavioral and emotional domains across the current state of the literature. A second challenge is the focus of many studies on targeted age groups of children or adolescents. Of the seven reviewed studies, five focused on singular age groups including children varying in age from 2 to 14 years (32, 33, 35–37). Even the two studies that included a wider age range restricted their focus to relatively small developmental periods (31, 34). The lack of follow-up across a broader age range limits knowledge regarding the developmental maturation of behavioral and emotional difficulties and identification of potential critical periods of psychopathological development or of possible age-related increases or decreases in risk for psychiatric problems (8, 38). Third, existing research has focused primarily on children and adolescents born EPT/ELBW (31, 34–37). Despite EPT/ELBW infants being at greater risk for morbidity, this sub-category of prematurity only represents a relatively small proportion of all preterm births. In fact, late preterm births account for approximately 75% of all preterm births (39). Although even this lower-risk subset of the preterm population experiences adverse consequences (40), only one of the studies reviewed above examined the preterm behavioral phenotype in these children (35). Finally, existing studies have relied on a symptom-based approach as opposed to more comprehensive clinical diagnoses, as seen by only one reviewed study including diagnostic assessment (37). Researchers have over-utilized both generic behavioral or emotional screening measures such as the SDQ and unitary diagnosis-specific measures such as the Conners’ Parent Rating Scale. Reliance on these measures limits our understanding of the full extent of psychopathology and potential developmental differences in clinical symptomatology.

**Table 2:** Outcome measures of included studies investigating the symptomatology of the preterm behavioral phenotype.

Reference	Measure	Type of Assessment
Loe et al. (31)	Child Behavior Checklist	Screening
Taylor et al. (32)	Adolescent Symptom Inventory-4 Youth’s Inventory-4	Screening Screening
Korzeniewski et al. (33)	Social Responsiveness Scale	Screening
Samuelsson et al. (34)	Child Behavior Checklist (parent) and Teacher Report Form Conners’ Parent Rating Scale and Teacher Report Form	Screening Screening
Johnson et al. (35)	Brief Infant-Toddler Social and Emotional Assessment Modified Checklist for Autism in Toddlers	Screening Screennig
Burnett et al. (36)	Strengths and Difficulties Questionnaire	Screening
Lean et al. (37)	Child Behavior Checklist Conners’ Parent Rating Scale and Teacher Report Form Social Responsiveness Scale Preschool Age Psychiatric Assessment Autism Diagnostic Observation Schedule	Screening Screennig Screening Diagnostic Diagnostic

A prevailing limitation of the existing literature is that the manifestation of the preterm behavioral phenotype is very rarely described in relation to underlying etiological mechanisms. Therefore, it remains unclear if the unique comorbid nature of the phenotype has a distinct neurological or psychosocial basis precipitating its development.

The impetus to conduct more research on neurological alterations associated with the preterm behavioral phenotype is growing with the increase in magnetic resonance imaging studies of preterm samples. In the preterm population, ADHD has been associated with reduced volumes in total cerebral and frontal areas (19, 41), ventriculomegaly (42), and white matter injury (43). ASD and social–communicative problems have also been associated with brain abnormalities as defined by reductions in total cerebral volume (44) and in volumes of the frontal, occipital, and limbic regions (45, 46), as well as periventricular leukomalacia (47), and other white matter abnormalities (44). Similarly, a potential explanation for increased anxiety and internalizing problems in the preterm population include white matter abnormalities (43) and reduced volume and connectivity in the amygdala (48–51). While there is support for neurological correlates of ADHD, ASD, and anxiety in the preterm population independently, there is little evidence to support brain mechanisms of comorbid psychopathology. Endorsing this idea, only one of the seven studies included in this review included neurological investigations of comorbid ADHD and anxiety (31). Thus, research is required to identify the brain regions, neural circuits, and patterns of connectivity implicated in the preterm behavioral phenotype.

Maternal mental health is another factor that may underlie the unique development of comorbid psychopathology in preterm children (52). Mothers of preterm infants are significantly more likely than mothers of full-term infants to experience postpartum depression (53, 54), anxiety, and post-traumatic stress (55–57). A recent longitudinal study by Priel et al. (58) reported that maternal depression markedly increased the risk for childhood psychopathology at both 6 and 10 years of age in association with heightened oxytocin levels of the child. Findings imply the existence of underlying neurobiological mechanisms that account for the relationship between maternal mental health and adverse child psychiatric outcomes. While the sample for this study was not limited to preterm children, more pronounced effects of maternal mental health on behavior may be found in this high-risk population. In support of this possibility, maternal depression has been associated with higher psychiatric problems in preschool and school-age preterm children (40, 59), with lower levels of depression enhancing childhood resilience against psychiatric and neurodevelopmental problems (37).

The primary mechanisms of the preterm birth experience and the resultant maternal mental health can both negatively impact parenting behavior and mother–child relationships (60, 61), which may further contribute to the unparalleled psychiatric profile of preterm children. A recent meta-analysis by Bernard et al. (54) of 48 studies found that mothers of preterm infants with higher levels of depression were less sensitive towards their infants than mothers with lower levels of depression. In addition, the lack of maternal sensitivity within the first year of life is associated with altered hippocampal networks (62), implicating potential effects on infant bonding and attachment (60, 63). These disruptions to secure mother–infant relationships pose risks for later childhood psychopathology (59), including internalizing and externalizing behaviors (64). The unique risks of preterm birth for altered brain development, problems in maternal mental health, and parenting behaviors may help explain the exclusive and specific comorbidities of psychopathology observed in this high-risk population. However, currently, these mechanisms remain primarily speculative and pose large gaps in the current understanding of the preterm behavioral phenotype, warranting further exploration.

Eight systematic reviews and meta-analyses have been published since the preterm behavioral phenotype was proposed in 2011 (8, 12, 17, 20, 65–68), each of which synthesizes the findings of between 6 and 74 studies, often with a single-outcome focus. However, they offer limited information on the nature of the phenotype. While these meta-analyses provide useful data on the prevalence of various psychiatric conditions, little information is available on psychiatric comorbidity. The current review addresses this gap in knowledge by summarizing existing findings on behavioral comorbidity. Conclusions drawn from eligible studies may nevertheless be limited by the strict inclusion criteria applied in identifying relevant research. Specifically, the current review considered studies for inclusion only if they explicitly referred to the preterm behavioral phenotype. Although this decision was made to enable the convergence and characterization of available literature on this proposed phenotype, some studies relevant to the understanding of comorbidity of behavior problems in preterm children were likely excluded.

Enhancing understanding of the preterm behavioral phenotype will require further investigation of the neonatal, neural, and psychosocial antecedents of the types and comorbidity of behavioral and emotional problems in preterm children. Longitudinal studies are needed to address questions regarding influences on developmental precursors of psychiatric disorders and identification of risk and resilience factors. Finally, critical review of the current *DSM-IV/V*–based conceptualization of the preterm behavioral phenotype is needed in view of the vast variability in the preterm population in etiology, symptom presentation, and comorbidity.

## Conclusions

The objective of this state-of-the-art review was to comprehensively synthesize and describe the studies characterizing the preterm behavioral phenotype since conceptualization in 2011. Findings confirm the suggestion that children born preterm are at significantly increased risk for symptoms of ADHD, ASD, and anxiety disorders. Although there is some additional support for comorbidity of these three symptom clusters, few studies have investigated the extent to which these clusters occur conjointly as a unitary phenotype, in isolation, or in combination with other behavioral, cognitive, or learning impairments. The pattern of comorbidities associated with preterm birth has implications for neurodevelopmental assessment of children born preterm and for addressing their clinical needs. As profiles of impairment may vary with age, knowledge of comorbidities may be useful in improving early identification of children at the highest risk for ongoing psychiatric disorders. Identification of profiles of comorbidity may also be useful in guiding more individualized approaches to intervention.

## Author Contributions

The corresponding author, SB, had full access to all of the study data and is primarily accountable for all aspects of the work, including the decision to submit for publication. GF conceptualized and designed the review protocol, acquired data, interpreted the results, drafted and revised the initial manuscript, and approved the final manuscript as submitted. GT substantially contributed to the oversight of the conceptualization of the review, interpreted the results, critically reviewed and revised the initial manuscript, and approved the final manuscript as submitted. SB acquired funds and resources, conceptualized the review, supervised the designing of the review protocol and data acquisition, interpreted the results, critically reviewed and revised the initial manuscript, and approved the final manuscript as submitted.

## Funding

This work was supported by a Mater Foundation Principal Research Fellowship to SB and the University of Queensland Research Training Program and Frank Clair scholarships to GF. The funding sources had no role in the writing of the manuscript or in the decision to submit it for publication.

## Conflict of Interest

The authors declare that the research was conducted in the absence of any commercial or financial relationships that could be construed as a potential conflict of interest.

## Abbreviations

ADHD, attention-deficit/hyperactivity disorder; ASD, autism spectrum disorder; CBCL, Child Behavior Checklist; *DSM-III-R, Diagnostic and Statistical Manual of Mental Disorders Third Edition Revised; DSM-IV*, *Diagnostic and Statistical Manual of Mental Disorders, Fourth Edition*; ELBW, extremely low birth weight; EPT, extremely preterm; ICD-10, International Classification of Diseases 10^th^ Revision; LBW, low birth weight; LMPT, late to moderate preterm; SDQ, Strengths and Difficulties Questionnaire; SRS, Social Responsiveness Scale; VPT, very preterm.
